# The Impact of the Human Papillomavirus Vaccine on High-Grade Cervical Lesions in Urban and Rural Areas: An Age–Period–Cohort Analysis

**DOI:** 10.3390/cancers13164215

**Published:** 2021-08-21

**Authors:** Jaimie Z. Shing, Alicia Beeghly-Fadiel, Marie R. Griffin, Rachel S. Chang, Staci L. Sudenga, James C. Slaughter, Manideepthi Pemmaraju, Edward F. Mitchel, Pamela C. Hull

**Affiliations:** 1 Division of Epidemiology, Department of Medicine, Vanderbilt University Medical Center, Nashville, TN 37232, USA; alicia.beeghly@vumc.org (A.B.-F.); staci.sudenga@vumc.org (S.L.S.); 2 Department of Health Policy, Vanderbilt University Medical Center, Nashville, TN 37232, USA; marie.griffin@vumc.org (M.R.G.); manideepthi.pemmaraju@vumc.org (M.P.); ed.mitchel@vumc.org (E.F.M.); 3 School of Medicine, Vanderbilt University, Nashville, TN 37232, USA; rachel.s.chang@vanderbilt.edu; 4 Department of Biostatistics, Vanderbilt University Medical Center, Nashville, TN 37232, USA; james.c.slaughter@vumc.org; 5 Department of Behavioral Science, University of Kentucky Markey Cancer Center, Lexington, KY 40504, USA; pam.hull@uky.edu

**Keywords:** human papillomavirus, vaccine impact, high-grade cervical lesions, cervical premalignant lesions, international classification of diseases, urbanicity, metropolitan statistical area

## Abstract

**Simple Summary:**

Human papillomavirus (HPV) vaccination disparities between urban and rural regions may moderate the vaccine’s impact on reducing cervical precancer (CIN2+) and cancer incidence. We assessed population-level trends in CIN2+ incidence (2008–2018) in urban and rural areas among Medicaid-enrolled women aged 18–39 years in Tennessee, United States. A sub-group analysis among women screened for cervical cancer was conducted to control for changing screening trends. CIN2+ incidence among young women aged 18–20 and 21–24 years, who most likely benefited from the HPV vaccine, declined similarly between urban and rural areas, although significant declines began earlier in urban versus rural areas. Our results suggest evidence of HPV vaccine impact regardless of urbanicity but demonstrate lagged impact in rural areas. These findings emphasize the importance of reducing barriers to HPV vaccination, particularly in rural areas, to improve the reduction of cervical precancer and cancer incidence, toward the World Health Organization’s goals of eliminating cervical cancer.

**Abstract:**

Disparities in human papillomavirus (HPV) vaccination exist between urban (metropolitan statistical areas (MSAs)) and rural (non-MSAs) regions. To address whether the HPV vaccine’s impact differs by urbanicity, we examined trends in cervical intraepithelial neoplasia grades 2 or 3 and adenocarcinoma in situ (collectively, CIN2+) incidence in MSAs and non-MSAs among Tennessee Medicaid (TennCare)-enrolled women aged 18–39 years and among the subset screened for cervical cancer in Tennessee, United States. Using TennCare claims data, we identified annual age-group-specific (18–20, 21–24, 25–29, 30–34, and 35–39 years) CIN2+ incidence (2008–2018). Joinpoint regression was used to identify trends over time. Age–period–cohort Poisson regression models were used to evaluate age, period, and cohort effects. All analyses were stratified by urbanicity (MSA versus non-MSA). From 2008–2018, 11,243 incident CIN2+ events (7956 in MSAs; 3287 in non-MSAs) were identified among TennCare-enrolled women aged 18–39 years. CIN2+ incident trends (2008–2018) were similar between women in MSAs and non-MSAs, with largest declines among ages 18–20 (MSA average annual percent change (AAPC): −30.4, 95% confidence interval (95%CI): −35.4, −25.0; non-MSA AAPC: −30.9, 95%CI: −36.8, −24.5) and 21–24 years (MSA AAPC: −14.8, 95%CI: −18.1, −11.3; non-MSA AAPC: −15.1, 95%CI: −17.9, −12.2). Significant declines for ages 18–20 years began in 2008 in MSAs compared to 2010 in non-MSAs. Trends were largely driven by age and cohort effects. These patterns were consistent among screened women. Despite evidence of HPV vaccine impact on reducing CIN2+ incidence regardless of urbanicity, significant declines in CIN2+ incidence were delayed in non-MSAs versus MSAs.

## 1. Introduction

The current nonavalent human papillomavirus (HPV) vaccine can prevent up to 90% of cervical cancer cases [1]. Despite being vaccine-preventable, cervical cancer remains the fourth most common incident cancer in women worldwide, causing over 300,000 cervical cancer-related deaths annually [2]. In the United States (US) and other high-income countries, studies have demonstrated reductions in intermediate endpoints for cervical cancer, such as high-grade cervical lesions, including cervical intraepithelial neoplasia (CIN) grades 2 and 3, and adenocarcinoma in situ (together referred to as CIN2+) [3].

In the US, the HPV vaccine is covered for all children up to age 18 years under the federal Vaccines for Children program. While studies have documented overall declines in HPV-related adverse health outcomes among younger age groups who were most likely to have benefited from the introduction of the vaccine, disparities in HPV vaccination exist between urban and rural geographical regions [4,5,6,7,8], raising concern that the timing or magnitude of vaccine impact may differ for these populations. Specifically in the US, HPV vaccine initiation among adolescents aged 13–17 years in urban areas, known as metropolitan statistical areas (MSAs), increased from 49% to 74% from 2013 to 2019, compared to 37% to 64% in rural areas (non-MSAs) [7,9]. Despite increasing adolescent HPV vaccination within urban and rural areas over time, annual vaccination coverage has significantly lagged behind in rural areas compared to urban areas [7].

Similar geographic disparities have also been demonstrated among adults, with 42% lower odds of HPV vaccine initiation for adults aged 18–26 years in rural areas compared to urban areas across eight US states [8]. These geographic differences may be attributed to rural areas having more barriers to vaccination, including lack of health care access, lack of knowledge and awareness of HPV and its link to cancer, increased negative community messaging regarding the vaccine, and more prevalent religious and cultural beliefs that may not support vaccination [10]. Given these large geographic disparities in both adolescent and adult HPV vaccination, examining whether urbanicity has modified the vaccine’s impact on reducing HPV-related outcomes is important for informing HPV vaccination guidelines and public health interventions to improve vaccination rates.

Low- and middle-resource countries have added barriers to HPV vaccination, including lack of vaccine availability due to the high cost of the vaccine, difficulties with implementing routine adolescent immunization programs, and competing health priorities [11]. Thus, examining trends in HPV-related health outcomes among lower resource settings, such as rural regions in the US, can provide valuable information on the HPV vaccine’s impact on reducing HPV-related health outcomes even when vaccination rates are low.

A few studies have examined trends in HPV-associated health outcomes by urbanicity [12,13,14,15]; of these, most have focused on the vaccine’s impact on reducing anogenital warts [13,14,15]. Only one study to date has assessed trends in CIN2+ incidence by urbanicity, reporting significant declines in CIN2+ incidence from 2008 to 2011 among women aged 21–24 years in both urban and rural counties in Connecticut [12]. However, this study did not examine CIN2+ trends by urbanicity for other age groups and did not control for possible changes in cervical cancer screening over time. To better understand the HPV vaccine’s impact on CIN2+ by urbanicity, we examined temporal trends in CIN2+ incidence, including age, period, and birth cohort effects, from 2008 through 2018, in urban and rural areas in Tennessee among (1) women aged 18–39 years enrolled in the Tennessee Medicaid (TennCare) program and (2) the subset of women who were screened for cervical cancer to control for changes in screening rates over time.

## 2. Methods

### 2.1. Study Population

The Medicaid program is a federal public insurance program in the US that covers low-income individuals. In the state of Tennessee, the Medicaid program is called TennCare. We used TennCare billing claims data to identify women aged 18–39 years who were enrolled in TennCare from 2008 to 2018. For the subset of screened women, we identified TennCare-enrolled women who were screened for cervical cancer at least once during any given year using billing codes for:

- an HPV screening examination: International Classification of Diseases 9th revision (ICD-9) code V73.81 or International Classification of Diseases 10th revision (ICD-10) code Z11.51; or
- a Papanicolaou test: ICD-9 codes V72.31, V72.32, V76.2, V76.47, 795.06, 91.46, or ICD-10 codes Z01.411, Z01.419, Z01.42, Z12.4, Z12.72, R87.614, or Current Procedural Terminology (CPT) codes 88141-88145, 88147-88148, 88150-88158, 88164-88167, 88174-88175, or Healthcare Common Procedure Coding System codes P3000-P3001, G0101, G0123-G0124, G0141, G0143-G0145, G0147-G0148, Q0091; or
- an HPV deoxyribonucleic acid (DNA) test: ICD-9 codes 795.05, 795.09, or ICD-10 codes R87.10, R87.820, or CPT codes 87620-87622, 87623-87625.

To examine CIN2+ trends by urbanicity, women with missing data on residence were excluded. Urbanicity was categorized by county of residence using the MSA definitions and boundaries set by the US Census Bureau [16]. Urban areas, or MSAs, were counties with at least one area with a population of at least 50,000 persons, while all other counties were considered rural areas (non-MSAs) [16]. This study was considered public health surveillance (i.e., not human research) by the Institutional Review Boards at Vanderbilt University and the Tennessee Department of Health. This research activity was reviewed and approved by the Tennessee Department of Finance and Administration Division of TennCare.

### 2.2. Incident CIN2+ Event Definition

Incident CIN2+ events, including cervical intraepithelial lesions grades 2 and 3, and adenocarcinoma in situ, were identified by a validated claims-based model using billing codes among women with cervical diagnostic procedures who were consecutively enrolled in TennCare for at least one year from their diagnostic procedure date, as previously described in detail [17]. Briefly, among women with cervical diagnostic procedures, we derived predicted probabilities of CIN2+ using billing codes that indicated:

- relevant diagnoses: CIN2+ tissue, non-specific CIN, high-grade squamous intraepithelial lesion cytology, CIN grade 1 tissue, low-grade squamous intraepithelial lesion cytology, and atypical squamous cells of undetermined significance; and/or
- cervical screening: HPV screening examination, Papanicolaou test, and HPV DNA test; and/or
- relevant procedures: cervical treatment procedures and cervical or vaginal biopsies.

All corresponding billing codes have been provided previously [17]. For the subset of women who were screened for cervical cancer, CIN2+ events were counted if the screening date was within one year of the diagnostic procedure date. Incident CIN2+ events were model-identified events that did not have another CIN2+ event for at least one year prior to the diagnostic procedure date. Incident CIN2+ events in MSAs and non-MSAs were only counted if the woman resided in an MSA or non-MSA county, respectively, on the date of their cervical diagnostic procedure.

### 2.3. Denominator and Rates

Annual person-years for each age group (18–20, 21–24, 25–29, 30–34, and 35–39 years) were estimated by counting the total number of women who were enrolled in TennCare on July 1 of each year with at least one year of consecutive enrollment. For example, total person-years for 2008 comprised TennCare-enrolled women who were continuously enrolled between 1 July 2007, and 1 July 2008. Screened person-years included the subset of total women who had a least one cervical cancer screening code during the year prior to July 1 of each year. Only women residing in an MSA or non-MSA county on July 1 of each year were counted toward the person-time estimation for MSA and non-MSA populations, respectively. Annual CIN2+ incidence rates per 100,000 person-years were calculated by dividing the total number of women meeting the incident CIN2+ event definition by the estimated person-time for each year and age group among all women and those residing in MSA and non-MSA counties and then multiplying by 100,000.

### 2.4. Joinpoint Trend Analyses

We identified CIN2+ incident trends and significant changes in CIN2+ trends (i.e., changes in slope) over time using the Joinpoint Desktop Software version 4.5.01 (National Cancer Institute, Bethesda, MD, USA), which calculated average percent changes (APCs, beta coefficients for each trend) and average annual percent changes (AAPCs, weighted averages of APCs) from 2008 to 2018, by urbanicity [18]. Using grid search and permutation tests, we allowed for a maximum of two joinpoints detected per model with uncorrelated errors to determine the best fit log-linear models. Using a two-sided alpha threshold of 0.05, 95% confidence intervals (CI) that excluded 0 were considered statistically significant.

### 2.5. Age–Period–Cohort Analyses

Age, period, and birth cohort effects were evaluated using the Clayton and Schiffler modeling approach for age–period–cohort analyses [19]. Age effects are differences in biological or social processes linked to maturation, such as age-associated risk factors for CIN2+ (e.g., number of lifetime sexual partners, condom use, and prevalence of high-risk HPV-type infections). Period effects refer to environmental factors that impact all ages. Cohort effects are historical differences between groups who were born in different eras, such as the availability of the HPV vaccine for younger ages and ineligibility for older ages when the vaccine was first introduced.

The Clayton and Schiffler model building process begins with an age model, then adds a “drift” parameter (i.e., the sum of the linear period and cohort effects) [19]. Derivatives of the drift parameter are estimated and regressed on period and cohort to estimate their effects on trends [19]. The following sub models were derived: (1) age, (2) age–drift, (3) age–cohort, (4) age–period, and (5) age–period–cohort. The general multiplicative formula for the age–period–cohort models was based on Poisson regression to derive incidence rates (log(λ |A, P, C)) at age (A) in a period (P) for persons in birth cohort (C) using the following equation:

log(λ |A, P, C)): *f*(A) + *g*(P) + *h*(C), (1)

where A, P, and C represent the mean age, period, and birth cohort for the observational units, respectively, and *f*, *g*, and *h*, represent the functions for each effect [20]. Synthetic birth cohort groups were calculated by subtracting the midpoint of each age group (18–20, 21–24, 25–29, 30–34, and 35–39 years) from each one-year period (2008, 2009, 2010, 2011, 2012, 2013, 2014, 2015, 2016, 2017, and 2018).

Because of the linear dependency between age, period, and cohort effects (C=P-A), the simultaneous linear effects of all three effects cannot be estimated; therefore, any parameterization of the age–period–cohort model included two fixed levels and one slope among the three functions [19]. We parameterized our models based on the maximum likelihood of the age–period–cohort model, considering age effects as incidence rates for the reference period (2008) and period effects as rate ratios relative to the reference period (2008) [20]. Cohort effects were constrained to be 0 on average with 0 slope and therefore interpreted as rate ratios relative to the age–period predictions (i.e., residual rate ratios) [20]. We estimated annual percent changes (EAPC), or the overall linear trends, from the net drift in the age–drift models.

Model goodness-of-fit was examined using residual deviance statistics. Using the Clayton and Schiffler approach [19], model fit was assessed for each sub model, comparing each iterative model to the primary model of age alone by sequentially adding cohort and period effects to determine whether these added parameters significantly improved model fit. Then, model fit was deductively assessed by iteratively removing parameters and testing whether this significantly reduced model fit. We tested for significant differences in residual deviance of each pairwise comparison using chi-squared tests. All age–period–cohort analyses were conducted using the apc.fit function from the Epi package in R (version 3.6.2) [20]. *p*-values <0.05 were considered statistically significant.

## 3. Results

### 3.1. Age-Specific Trends in CIN2+ Incidence

Between 2008 and 2018, we identified 7956 incident CIN2+ events in MSA counties, compared to 3287 incident CIN2+ events in non-MSA counties (total: 11,243) (Table 1).

**Figure 1:**
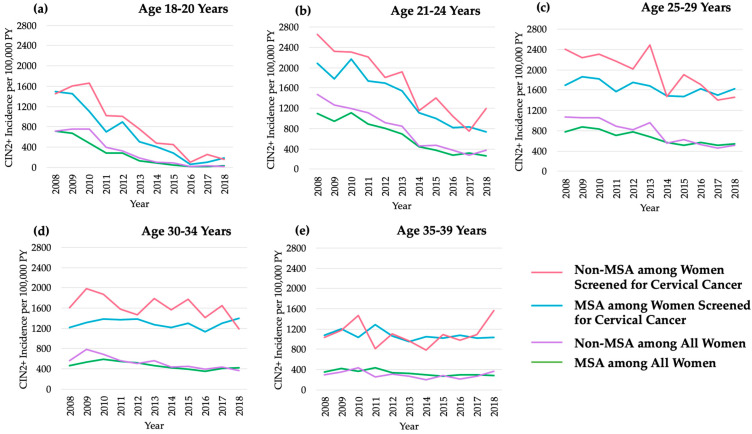
Annual CIN2+ incidence per 100,000 person-years among all women enrolled in Tennessee Medicaid and the subset of women screened for cervical cancer who resided in MSAs versus non-MSAs aged: (**a**) 18–20 years; (**b**) 21–24 years; (**c**) 25–29 years; (**d**) 30–34 years; (**e**) 35–39 years. CIN2+: cervical intraepithelial neoplasia grades 2 and 3 and adenocarcinoma in situ; MSA: metropolitan statistical area; PY: person-years.

Of the total number of events, 10,540 (94%) women (7470 MSA; 3070 non-MSA) had a cervical screening code identified in the year prior to their incident event. Among women residing in MSAs, CIN2+ incidence significantly declined from 2008 to 2018 for those aged 18–20 years (AAPC: −30.4; 95% CI: −35.4, −25.0), 21–24 years (AAPC: −14.8; 95% CI: −18.1, −11.3), 25-29 years (AAPC: −5.3; 95% CI: −7.1, -3.6), and 35–39 years (AAPC: −3.9; 95% CI: −5.8, −1.9) (Table 2, Figure 1).

However, after restricting to screened women, declines in CIN2+ were only observed for the youngest three age groups. Among women residing in non-MSAs, CIN2+ incidence significantly declined for those aged 18–20 years (AAPC: −30.9; 95% CI: −36.8, −24.5), 21–24 years (AAPC: −17.9, 95% CI: −17.9, −12.2), 25–29 years (AAPC: −8.8; 95% CI: −11.3, −6.3), and 30–34 years (AAPC: −6.2; 95% CI: −8.5, −3.8). Again, after restricting to women who were screened for cervical cancer, significant declines were only observed for the three youngest age groups.

Several Joinpoint-detected inflections (e.g., time points where there are significant changes in slopes across time periods) were identified (Table 3). Among women residing in MSAs, inflections were only observed for those aged 30–34 years, with significant increases in CIN2+ incidence from 2008 to 2010 (APC: 13.3; 95% CI: 1.5, 26.4), followed by significant decreases from 2010–2016 (APC: −8.0; 95% CI: −9.9, −6.1). This pattern was mirrored, yet less pronounced and non-significant, among screened women aged 30–34 years who resided in MSAs. Among women residing in non-MSAs, an inflection was only observed among those aged 18–20 years, with stable trends from 2008 to 2010, followed by significant declines in CIN2+ incidence from 2010 to 2018 (APC: −37.0; 95% CI: −43.4, −29.9). This pattern was similar to that of screened women aged 18–20 years who resided in non-MSAs.

### 3.2. Descriptive Age, Period, Cohort Effects

**Figure 2:**
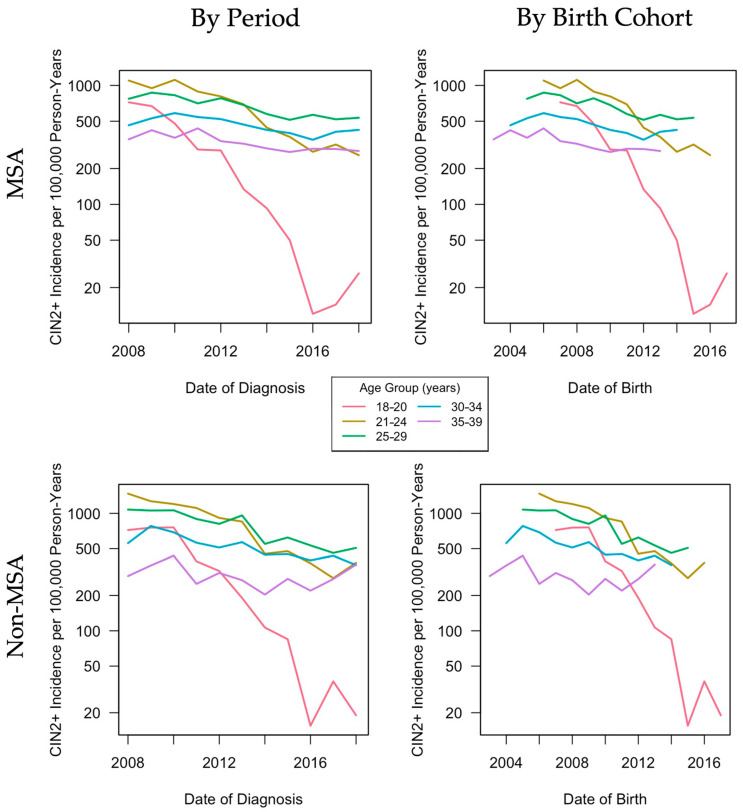
Age-group-specific CIN2+ incidence per 100,000 person-years by period and birth cohort among all women enrolled in Tennessee Medicaid. CIN2+: cervical intraepithelial neoplasia grades 2 and 3 and adenocarcinoma in situ; MSA: metropolitan statistical area.

**Figure 3:**
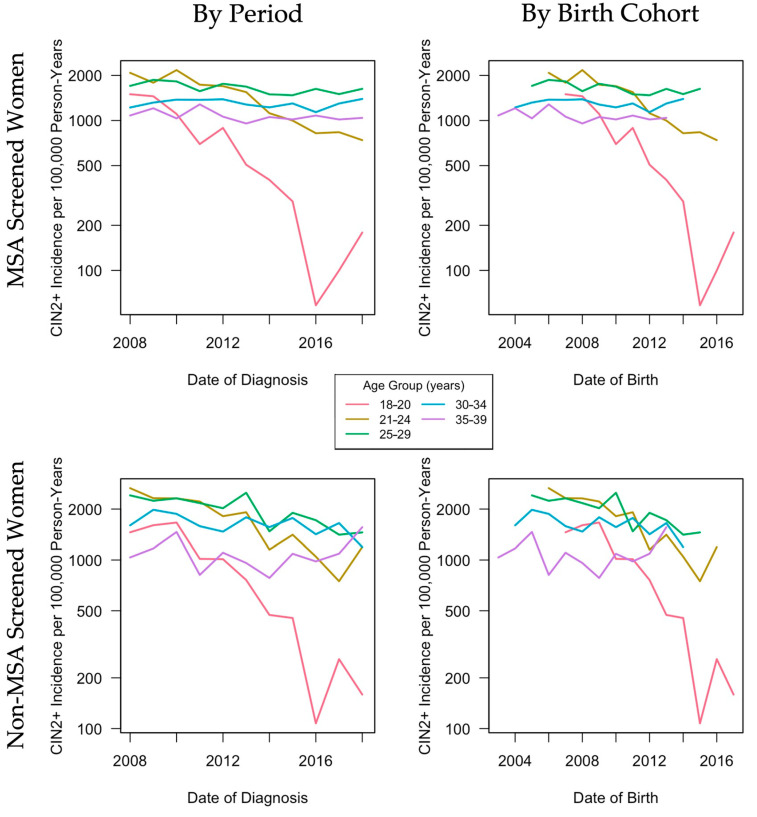
Age-group-specific CIN2+ incidence per 100,000 person-years by period and birth cohort among the subset of women enrolled in Tennessee Medicaid who were screened for cervical cancer. CIN2+: cervical intraepithelial neoplasia grades 2 and 3 and adenocarcinoma in situ; MSA: metropolitan statistical area.

Women residing in MSAs showed similarities in CIN2+ incidence rates by period and birth cohort compared to women residing in non-MSAs (Figure 2). Patterns were similar in that young women aged 18–20, 21–24, and 25–29 years had higher CIN2+ incidence at baseline (2008) compared to older women, with the highest rates among women aged 21–24 years (1098.7/100,000 person-years in MSAs and 1471.7/100,000 person-years in non-MSAs). The most drastic changes in CIN2+ rates were in the youngest age group (18–20 years) from 720.5/100,000 person-years in 2008 to 26.3/100,000 person-years in 2018 among MSAs and 720.5/100,000 person-years in 2008 to 19.0/100,000 person-years in 2018 among non-MSAs. Rates of decline varied by age group. For younger age groups, women who were born later had lower CIN2+ incidence rates. This pattern was also observed in screened women (Figure 3).

### 3.3. Age, Period, and Cohort Effects in Poisson Regression Models

Age–period–cohort Poisson regression models indicated decreasing CIN2+ incidence from 2008 to 2018 for both MSAs (EAPC: 0.90%/year) and non-MSAs (EAPC: 0.89%/year) (Table 4). Significant improvements in model fit were found when adding drift (i.e., the overall linear trend in CIN2+ incidence), period, and cohort effects (*p* < 0.001). The best-fitting model included all three effects (age–period–cohort), indicated by the lowest residual deviance, for women residing in MSAs (residual deviance: 662.6) and non-MSAs (residual deviance: 410.9). Model comparisons demonstrated notably larger cohort than period effects (change in deviance for nonlinear cohort effects versus nonlinear period effects: 562.3 versus 51.3 [MSA], 203.5 versus 19.8 [non-MSA]).

Among screened women, age–cohort and age–period–cohort models had similar goodness-of-fit, with slightly better fit for age–period–cohort models (residual deviance for age–cohort versus age–period–cohort: 463.6 versus 436.6 (MSA), 301.1 versus 296.9 (non-MSA)) (Table 4).

Among screened women residing in MSAs, cohort effects were larger than period effects (change in deviance for nonlinear cohort effects versus nonlinear period effects: 266.3 versus 15.6). Among screened women residing in non-MSAs, nonlinear period effects and period effects adjusted for cohort effects were not significant (*p* > 0.05); however, the drift, nonlinear cohort effects, and cohort effects adjusted for period effects were significant (*p* < 0.001).

**Figure 4:**
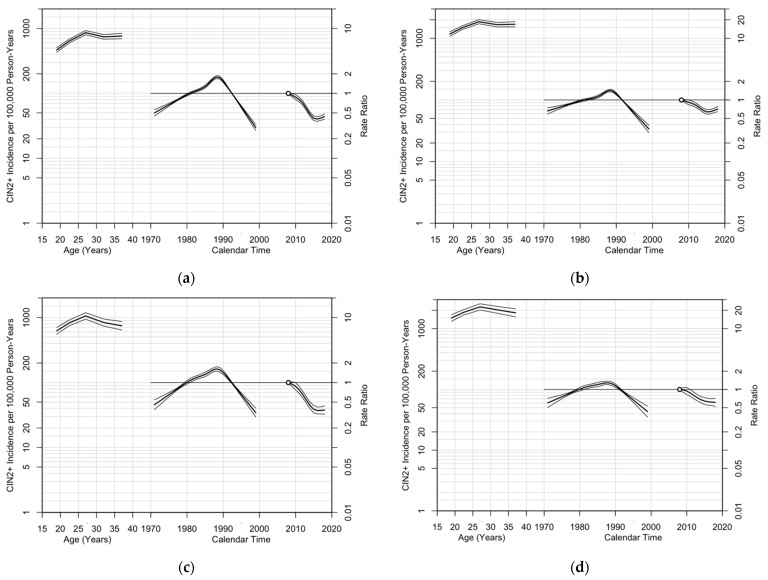
Age, cohort, and period effects ^1^ among (**a**) all women enrolled in Tennessee Medicaid residing in MSAs; (**b**) the subset of women screened for cervical cancer residing in MSAs; (**c**) all women enrolled in Tennessee Medicaid residing in non-MSAs; (**d**) the subset of women screened for cervical cancer residing in non-MSAs. CIN2+: cervical intraepithelial neoplasia grades 2 and 3 and adenocarcinoma in situ; MSA: metropolitan statistical area. ^1^ Each plot’s horizontal axis is divided into two parts: age, ranging from 15–40 years (left), and calendar time, ranging from 1970–2020 (right). Each plot contains two vertical axes: CIN2+ incidence per 100,000 person-years (left) and rate ratios (right), and three sets of curves: age effects, interpretable as cross-sectional CIN2+ incidence rates per 100,000 women at risk for the reference period, 2008, adjusted for cohort effects, with corresponding 95% confidence intervals (left), cohort effects, constrained to be 0 on average with 0 slope, interpretable as rate ratios relative to the age–period predictions (i.e., residual rate ratios) with corresponding 95% confidence intervals (middle), and period effects, interpretable as rate ratios relative to the reference period, 2008 (indicated by the hollow circle), with corresponding 95% confidence intervals (right).

For both women residing in MSAs and non-MSAs, age effects showed increasing CIN2+ incidence with increasing age among younger women until a peak of around age 27 years, followed by plateauing or decreasing CIN2+ incidence with increasing age among older women (Figure 4). Cohort effects demonstrated that women born between 1970 to 1988 experienced higher CIN2+ incidence with later years of birth, while women born after 1988 experienced lower CIN2+ incidence with later years of birth. Period effects demonstrated decreasing CIN2+ incidence from 2009 to the mid-2010s, and then a mild increasing curvature in the late 2010s. For screened women, CIN2+ incidence had similar, yet less steep and prominent, period effect patterns compared to all women.

## 4. Discussion

We examined temporal trends in CIN2+ incidence, including age, period, and cohort effects, among TennCare-enrolled women from 2008 to 2018, by urbanicity. In both MSAs and non-MSAs, our results demonstrated declining trends in CIN2+ incidence among women aged 18–39 years from 2008 onward, with the most drastic declines among young women aged 18–20 years and 21–24 years. As shown by the significant cohort and age effects, declines in CIN2+ were likely because of the HPV vaccine’s introduction in 2006 and the Advisory Committee on Immunization Practices’ recommendations for adolescent HPV vaccination, as well as changes in cervical cancer screening and management recommendations and aggressiveness of approach [21,22,23]. Although patterns and rates of decline in CIN2+ incidence were similar between women residing in MSAs and non-MSAs, significant declines were delayed until 2010 for women residing in non-MSAs, unlike in MSAs, which began in 2008. After restricting our analyses to women screened for cervical cancer to control for the confounding effects of changing screening rates over time, HPV vaccine impact was still evident, regardless of urbanicity.

Our age–period–cohort analyses indicated that trends in CIN2+ incidence were largely driven by cohort effects (i.e., historical factors associated with birth year) and age effects (i.e., social and biological variations associated with age), even after adjusting for period effects. Specifically, young women in more recent generations had lower rates of CIN2+ compared to young women born earlier. Cohort effects were likely due to generational differences in vaccine eligibility, vaccination behaviors, and screening recommendations in the US. When the Food and Drug Administration approved the first quadrivalent HPV vaccine in 2006 for females aged 9–26 years [21], older women were ineligible for the vaccine. Further, cervical cancer screening guidelines have changed. Historically in the US, screening was recommended within three years after sexual debut or at age 21 years (whichever occurred first); however, in 2012, screening in women younger than 21 years was no longer recommended, protecting adolescents and young women from unnecessary invasive gynecologic procedures that could increase their risk for cervical damage [23]. Changes in screening guidelines may have contributed to decreases in CIN2+ detection among younger women. Further, updated guidelines for the aggressiveness of follow-up approaches, such as frequency of screening, may have also contributed to less frequent screening and fewer colposcopies and biopsies to detect CIN2+ in screened women [23].

Our finding of significant age effects may be explained by age-associated social and biological factors regarding the HPV vaccine. Among age-eligible adults aged 18–26 years, HPV vaccination coverage in the US has been historically low, ranging from 22.1% to 39.9% for initiation and 13.8% to 21.5% for completion from 2013–2018 [24]. Additionally, HPV vaccination in women aged over 26 years may be less effective among those who have already been infected with HPV genotypes covered by the vaccine, creating biological barriers to preventing CIN2+ in this age group.

For urbanicity-stratified CIN2+ incidence by age group over time, we found similar patterns and evidence of HPV vaccine impact on reducing CIN2+ incidence in both MSAs and non-MSAs, despite varying HPV vaccination coverage by urbanicity. In Tennessee from 2016 to 2019, HPV vaccination in MSAs ranged from 66% to 69% for initiation and 46% to 47% for completion, compared to 46% to 53% for initiation and 25% to 34% for completion in non-MSAs [9]. A prior study in Connecticut also reported significant declines in CIN2+ incidence among young women in both urban and rural counties [12]. While HPV vaccination coverage rates are lower in rural communities than in urban communities, our results still demonstrated significant declines in CIN2+ in urban and rural settings. Additionally, despite varying HPV vaccination rates in urban and rural areas, a global-based meta-analysis reported similar genital HPV infection prevalence in urban (10%) and rural (11%) areas after the introduction of the HPV vaccine [25], suggesting that HPV infection rates are comparable regardless of urbanicity. This finding is corroborated by our prior work among TennCare-enrolled women, showing similar age-group-specific anogenital wart incidence, an HPV-associated outcome, by urbanicity [13].

Among women who were screened for cervical cancer, HPV vaccine impact was still evident in MSAs and non-MSAs, with similar declining CIN2+ incidence in young, screened women (aged 18–20 and 21–24 years) residing in MSAs and non-MSAs. However, in MSAs, significant declines were observed in screened women aged 25–29 years, while declines in non-MSAs for this age group were not significant. This may be due to improved accessibility of HPV vaccination in urban centers upon first release. Delays in HPV vaccination access and distribution within non-MSAs due to lack of healthcare sites, limited transportation, or other barriers may have contributed to a smaller initial impact of the HPV vaccine. However, similar declines in CIN2+ incidence in both MSAs and non-MSAs for younger cohorts indicate that non-MSAs were able to overcome initial barriers to HPV vaccination to have a comparable vaccine impact to MSAs. Further, age, period, and cohort effects were all significant among screened women residing in MSAs, while only age and cohort effects were significant for those residing in non-MSAs. However, because our sample size for women residing in MSAs was roughly double that of non-MSAs, we cannot rule out the possibility that differences between MSAs and non-MSAs were also due to differences in power and sample size.

Our study has limitations. The study represents a unique population of Tennessee Medicaid women; thus, results may not be generalizable to other geographical regions or to populations of higher socioeconomic status. For instance, Medicaid populations have very low cervical cancer screening rates compared to the general US population [26]. Additionally, due to the limitations of other variables that could potentially be associated with CIN2+ in the TennCare database, such as race/ethnicity and income level [27], our results did not consider these factors. Specifically, women of Black race and those with higher levels of poverty have been shown to have higher CIN2+ rates [27]; thus, these factors may impact CIN2+ trends by urbanicity. Furthermore, because this is an ecologic study, we were unable to examine individual-level vaccination data, but instead were able to account for both direct and indirect effects of the HPV vaccine. Lastly, we cannot exclude the likelihood that some of the variations in CIN2+ trends by urbanicity were due to differences in sample size between MSAs and non-MSAs, especially after restricting to women who were screened for cervical cancer.

Our study has notable strengths. This is the first study to describe CIN2+ incident trends by urbanicity using a validated claims-based model, demonstrating the applicability of utilizing claims data for CIN2+ surveillance research. Examining population-based CIN2+ trends in the US is costly and limited to populations with adequate surveillance of cervical biopsies through the New Mexico HPV Pap Registry [28] and the HPV Vaccine Impact Monitoring Project [29]. Utilizing claims data can be a more efficient way to monitor HPV vaccine impact; we were able to leverage TennCare claims data to detect vaccine impact on reducing CIN2+ incidence among Tennessee Medicaid enrollees, regardless of urbanicity. Further, this is the first US study to examine secular time trends in CIN2+ incidence using age–period–cohort models. Prior studies examining HPV vaccine impact on CIN2+ incidence have focused on evaluating overall linear trends using Joinpoint or pre-to-post vaccine era CIN2+ incidence using incidence rate ratios. We expand upon these prior studies by attempting to disentangle age, period, and cohort effects on CIN2+ trends using age–period–cohort models. However, due to the linear dependency of all three effects, the magnitude of each effect cannot be entirely isolated. Finally, our study has a large overall sample size, increasing the power of the results and reinforcing the validity of our findings. Given the power and unique low socioeconomic status of our Tennessee Medicaid study population, results may be translated to other low-income populations, particularly lower- and middle-resource countries, which are historically underrepresented, have a higher incidence of cervical cancer, and have increased barriers to cervical cancer screening. Greater insight into these understudied populations can be utilized to reduce socioeconomic disparities in cervical cancer, adding to the World Health Organization’s “90–70–90” global strategy to accelerate the elimination of cervical cancer, which includes 2030 targets for 90% of girls aged <15 years who are up-to-date on their HPV vaccination, 70% of women who are screened for cervical cancer by age 35 years, 90% of identified cervical precancers treated, and 90% of invasive cervical cancers managed [30].

## 5. Conclusions

In summary, we demonstrated significant declines in CIN2+ incidence in both MSAs and non-MSAs among TennCare-enrolled women, particularly in younger women who likely benefited from the HPV vaccine. CIN2+ trends were mostly driven by age and cohort effects, but effects in non-MSAs were delayed compared to MSAs, suggesting an impact of lower vaccination rates and delayed increases in vaccination in non-MSAs. These results emphasize the importance of reducing barriers to HPV vaccination in lower resource settings, such as rural regions, to promote cervical cancer elimination.

## Figures and Tables

**Table 1:** Annual age-group-specific CIN2+ incidence per 100,000 person-years among all women enrolled in Tennessee Medicaid and the subset of women screened for cervical cancer by urbanicity, 2008–2018.

Age (yrs)	MSA Residence					Non-MSA Residence				
	18–20	21–24	25–29	30–34	35–39	18–20	21–24	25–29	30–34	35–39
Among All Women										
**Total PY**	279,140	308,959	412,173	380,147	318,804	109,144	114,431	148,471	138,304	121,270
**CIN2+ Events**	640	1909	2664	1713	1030	312	847	1083	690	355
**Year**										
**2008**	720.5	1098.7	771.7	462.4	352.2	720.5	1471.7	1076.1	557.1	292.7
**2009**	669.3	946.9	869.1	529.2	420.0	755.5	1267.8	1056.5	779.7	360.1
**2010**	477.8	1113.3	827.0	584.6	362.9	758.7	1200.4	1061.4	689.1	436.0
**2011**	289.1	887.2	706.2	542.1	435.4	390.8	1110.6	895.0	561.9	251.2
**2012**	284.0	808.0	779.5	521.1	340.0	323.4	913.9	813.5	511.4	311.3
**2013**	133.7	692.3	683.4	468.2	323.2	190.6	851.0	959.4	568.1	270.1
**2014**	92.7	441.5	574.8	422.4	295.5	106.7	452.3	549.0	443.8	203.7
**2015**	49.9	369.2	513.7	397.5	275.4	84.7	476.1	622.0	450.1	277.4
**2016**	12.0	276.6	566.1	349.2	293.4	15.5	376.1	532.6	398.3	220.1
**2017**	14.4	318.4	518.8	407.6	291.9	37.0	281.0	460.1	435.4	276.2
**2018**	26.3	259.2	534.5	422.5	281.1	19.0	380.1	507.5	363.3	366.6
**Among Women Screened for Cervical Cancer**										
**Total PY**	75,181	123,842	153,229	122,514	88,209	29,593	44,909	51,356	40,178	30,174
**CIN2+ Events**	616	1798	2521	1591	944	305	783	1001	648	333
**Year**										
**2008**	1497.6	2081.3	1702.6	1221.0	1080.7	1456.2	2660.4	2411.1	1601.1	1035.2
**2009**	1451.8	1787.5	1867.3	1317.5	1206.8	1603.8	2319.1	2239.1	1979.6	1170.1
**2010**	1106.1	2170.3	1823.2	1377.9	1033.4	1663.3	2313.6	2315.5	1872.1	1462.7
**2011**	696.1	1734.7	1569.5	1373.9	1281.5	1015.4	2216.6	2169.7	1583.3	815.7
**2012**	892.1	1695.8	1758.4	1385.4	1060.7	1011.2	1814.1	2023.1	1470.2	1104.3
**2013**	506.9	1547.3	1683.0	1277.9	955.4	760.8	1914.8	2497.7	1787.3	959.9
**2014**	401.7	1118.5	1495.3	1223.7	1054.9	472.2	1151.0	1475.1	1562.5	782.5
**2015**	288.5	999.2	1474.9	1300.1	1016.9	452.7	1409.1	1899.6	1770.4	1088.4
**2016**	58.5	822.7	1626.2	1137.2	1079.2	107.4	1046.9	1718.0	1418.0	980.7
**2017**	99.9	835.8	1500.3	1298.8	1016.1	258.1	748.3	1409.6	1651.7	1089.7
**2018**	178.8	739.8	1627.1	1393.0	1041.0	159.1	1193.5	1453.7	1191.6	1561.0

CIN2+: cervical intraepithelial neoplasia grades 2 and 3, and adenocarcinoma in situ; MSA: metropolitan statistical area; PY: person-years.

**Table 2:** Average annual percent changes in age-group-specific CIN2+ incidence among all women enrolled in Tennessee Medicaid and the subset of women screened for cervical cancer, by urbanicity, 2008–2018.

	MSA Residence		Non-MSA Residence	
Age, Years	AAPC ^1^	95% CI	AAPC ^1^	95% CI
**Among All Women**				
**18–20**	−30.4 *	−35.4, −25.0	−30.9 *	−36.8, −24.5
**21–24**	−14.8 *	−18.1, −11.3	−15.1 *	−17.9, −12.2
**25–29**	−5.3 *	−7.1, −3.6	−8.8 *	−11.3, −6.3
**30–34**	−0.8	−2.6, 1.1	−6.2 *	−8.5, −3.8
**35–39**	−3.9 *	−5.8, −1.9	−1.5	−6.1, 3.2
**Among Women Screened for Cervical Cancer**				
**18–20**	−21.1 *	−26.1, −15.8	−19.8 *	−26.5, −12.4
**21–24**	−10.4 *	−13.2, −7.6	−10.0 *	−12.7, −7.1
**25–29**	−2.6 *	−2.9, −0.2	−4.9 *	−7.5, −2.3
**30–34**	1.3	−2.4, 5.2	−2.5	−5.0, 0.1
**35–39**	−1.1	−2.7, 0.5	1.1	−3.4, 5.8

AAPC: average annual percent change; CI: confidence interval; CIN2+: cervical intraepithelial neoplasia grades 2 and 3, and adenocarcinoma in situ; MSA: metropolitan statistical area. ^1^ Average annual percent changes are weighted averages of annual percent changes from 2008 to 2018. * Asterisks indicate statistical significance (*p* < 0.05).

**Table 3:** Annual percent changes in age-group-specific CIN2+ incidence among all women enrolled in Tennessee Medicaid and the subset of women screened for cervical cancer, by urbanicity, 2008–2018.

	MSA Residence				Non-MSA Residence			
	Inflection Year	Time Period	APC ^1^	95% CI	Inflection Year	Time Period	APC ^1^	95% CI
Among All Women								
**Age, Years**								
**18–20**	--	2008–2018	−30.4 *	−35.4, −25.0	2010	2008–2010	0.2	−29.4, 42.4
						2010–2018	−37.0 *	−43.4, −29.9
**21–24**	--	2008–2018	−14.8 *	−18.1, −11.3	--	2008–2018	−15.1 *	−17.9, −12.2
**25–29**	--	2008–2018	−5.3 *	−7.1, −3.6	--	2008–2018	−8.8 *	−11.3, −6.3
**30–34**	2010	2008–2010	13.3 *	1.5, 26.4	--	2008–2018	−6.2 *	-8.5, −3.8
	2016	2010–2016	−8.0 *	−9.9, −6.1				
		2016–2018	9.0	−0.2, 19.2				
**35–39**	--	2008–2018	−3.9 *	−5.8, −1.9	--	2008–2018	−1.5	−6.1, 3.2
**Among Women Screened for Cervical Cancer**								
**Age, Years**								
**18–20**	--	2008–2018	−21.1 *	−26.1, −15.8	2010	2008–2010	4.5	−26.2, 47.8
						2010–2018	−24.9 *	−32.5, −16.5
**21–24**	--	2008–2018	−10.4 *	−13.2, −7.6	--	2008–2018	−10.0 *	−12.7, −7.1
**25–29**	--	2008–2018	−2.6 *	−2.9, −0.2	--	2008–2018	−4.9 *	−7.5, −2.3
**30–34**	2010	2008–2010	7.5	−13.3, 33.3	--	2008–2018	−2.5	−5.0, 0.1
	2016	2010–2016	−2.8	−6.7, 1.2				
		2016–2018	8.4	−9.4, 29.7				
**35–39**	--	2008–2016	−1.1	−2.7, 0.5	--	2008–2018	1.1	−3.4, 5.8

APC: annual percent change; CI: confidence interval; CIN2+: cervical intraepithelial neoplasia grades 2 and 3 and adenocarcinoma in situ; MSA: metropolitan statistical area. ^1^ Annual percent changes were determined by the β-coefficient of the best fit log-linear model using a permutation test and Poisson variance for each time period detected by Joinpoint. * Asterisks indicate statistical significance (*p* < 0.05).

**Table 4:** Age–period–cohort models for CIN2+ incidence among all women enrolled in Tennessee Medicaid and the subset of women screened for cervical cancer, by urbanicity, 2008–2018.

	Goodness-of-Fit			Model Comparison					
	Residual df	Residual Deviance	*p*-Value	Model Comparison	Interpretation	Change in df	Change in Deviance	*p*-Value	EAPC (95% CI)
Among All Women									
**MSA**									0.90(0.90, 0.91)
**1. Age**	238	2006.75	--			--	--	--	
**2. Age–Drift**	237	1295.52	<0.001	2 versus 1	Trend (drift)	1	711.23	<0.001	
**3. Age–Cohort**	234	733.25	<0.001	3 versus 2	Nonlinear cohort effect	3	562.27	<0.001	
**4. Age–Period**	234	1244.25	<0.001	4 versus 2	Nonlinear period effect	3	51.27	<0.001	
**5. Age–Period**–**Cohort**	231	662.64	<0.001	5 versus 3	Period effect adjusted for cohort	3	70.60	<0.001	
				5 versus 4	Cohort effect adjusted for period	3	581.60	<0.001	
**Non-MSA**									0.89(0.88, 0.90)
**1. Age**	238	1080.50	--			--	--	--	
**2. Age–Drift**	237	639.10	<0.001	2 versus 1	Trend (drift)	1	441.40	<0.001	
**3. Age–Cohort**	234	435.57	<0.001	3 versus 2	Nonlinear cohort effect	3	203.53	<0.001	
**4. Age–Period**	234	619.29	<0.001	4 versus 2	Nonlinear period effect	3	19.81	<0.001	
**5. Age–Period–Cohort**	231	410.92	<0.001	5 versus 3	Period effect adjusted for cohort	3	24.66	<0.001	
				5 versus 4	Cohort effect adjusted for period	3	208.37	<0.001	
**Among Women Screened for Cervical Cancer**									
**MSA**									0.96(0.95, 0.96)
**1. Age**	238	891.14	--			--	--	--	
**2. Age–Drift**	237	729.89	<0.001	2 versus 1	Trend (drift)	1	161.25	<0.001	
**3. Age–Cohort**	234	463.58	<0.001	3 versus 2	Nonlinear cohort effect	3	266.30	<0.001	
**4. Age–Period**	234	714.29	<0.001	4 versus 2	Nonlinear period effect	3	15.60	0.001	
**5. Age–Period–Cohort**	231	436.61	<0.001	5 versus 3	Period effect adjusted for cohort	3	26.97	<0.001	
				5 versus 4	Cohort effect adjusted for period	3	277.68	<0.001	
**Non-MSA**									0.94(0.93, 0.96)
**1. Age**	238	499.55	--			--	--	--	
**2. Age–Drift**	237	383.80	<0.001	2 versus 1	Trend (drift)	1	1115.75	<0.001	
**3. Age–Cohort**	234	301.08	0.002	3 versus 2	Nonlinear cohort effect	3	82.72	<0.001	
**4. Age–Period**	234	380.60	<0.001	4 versus 2	Nonlinear period effect	3	3.20	0.362	
**5. Age–Period–Cohort**	231	296.86	0.002	5 versus 3	Period effect adjusted for cohort	3	4.21	0.239	
				5 versus 4	Cohort effect adjusted for period	3	83.73	<0.001	

CI: confidence interval; CIN2+: cervical intraepithelial neoplasia grades 2 and 3 and adenocarcinoma in situ; df: degrees of freedom; EAPC: estimated annual percent change; MSA: metropolitan statistical area.

## Data Availability

The data presented in the study were provided to Vanderbilt University Medical Center (VUMC) from the Division of TennCare of the Tennessee Department of Finance and Administration under a contract that does not permit VUMC to share the data with external parties. Researchers may request data from the Division of TennCare of the Department of Finance and Administration.
